# The role of WRKY transcription factors, *FaWRKY29* and *FaWRKY64*, for regulating Botrytis fruit rot resistance in strawberry (*Fragaria* × *ananassa* Duch.)

**DOI:** 10.1186/s12870-023-04426-1

**Published:** 2023-09-11

**Authors:** Man Bo Lee, Hyeondae Han, Seonghee Lee

**Affiliations:** 1 Present Address: Department of Plant Resources, College of Industrial Science, Kongju National University, Yesan, 32439 Korea; 2 Gulf Coast Research and Education Center, Institute of Food and Agricultural Science, University of Florida, Wimauma, FL 33598 USA

**Keywords:** Octoploid strawberry, *Botrytis cinerea*, Botrytis fruit rot, WRKY transcription factor

## Abstract

**Background:**

The cultivated strawberry (*Fragaria* × *ananassa* Duch.) is one of the most economically important horticultural crops worldwide. Botrytis fruit rot (BFR) caused by the necrotrophic fungal pathogen *Botrytis cinerea* is the most devasting disease of cultivated strawberries. Most commercially grown strawberry varieties are susceptible to BFR, and controlling BFR relies on repeated applications of various fungicides. Despite extensive efforts, breeding for BFR resistance has been unsuccessful, primarily due to lack of information regarding the mechanisms of disease resistance and genetic resources available in strawberry.

**Results:**

Using a reverse genetics approach, we identified candidate genes associated with BFR resistance and screened *Arabidopsis* mutants using strawberry isolates of *B. cinerea*. Among the five *Arabidopsis* T-DNA knockout lines tested, the mutant line with *AtWRKY53* showed the greatest reduction in disease symptoms of BFR against the pathogen. Two genes, *FaWRKY29* and *FaWRKY64*, were identified as orthologs in the latest octoploid strawberry genome, ‘Florida Brilliance’. We performed RNAi-mediated transient assay and found that the disease frequencies were significantly decreased in both *FaWRKY29-* and *FaWRKY64-*RNAi fruits of the strawberry cultivar, ‘Florida Brilliance’. Furthermore, our transcriptomic data analysis revealed significant regulation of genes associated with ABA and JA signaling, plant cell wall composition, and ROS in *FaWRKY29* or *FaWRKY64* knockdown strawberry fruits in response to the pathogen.

**Conclusion:**

Our study uncovered the foundational role of WRKY transcription factor genes, *FaWRKY29* and *FaWRKY64*, in conferring resistance against *B. cinerea*. The discovery of susceptibility genes involved in BFR presents significant potential for developing resistance breeding strategies in cultivated strawberries, potentially leveraging CRISPR-based gene editing techniques.

**Supplementary Information:**

The online version contains supplementary material available at 10.1186/s12870-023-04426-1.

## Background

The cultivated strawberry (*Fragaria* × *ananassa* Duch.) is one of the most economically important horticultural crops worldwide [1]. Strawberries are grown on 389,665 hectares and produced about 9.18 million tons worldwide in 2021 according to the Food and Agriculture Organization (https://www.fao.org/faostat/en/#data). The modern cultivated strawberry is an allo-octoploid (2*n* = 8*x* = 56) species. The first chromosome-scale genome assembly of *Fragaria* × *ananassa* was released from the cultivar ‘Camarosa’ in 2019 [2]. Recently, two high-quality haplotype-phased genomes from ‘Royal Royce’ [3] and ‘Florida Brilliance’ [4] are published and available at the Genome Database of Rosaceae (https://www.rosaceae.org/). The size of the phased octoploid strawberry genome is approximately 1.6 Gb containing 115,000 genes.

Strawberries are prone to various biotic stressors such as fungi, bacteria, viruses, and nematodes [5, 6]. Among these diseases, Botrytis fruit rot (BFR) disease caused by *Botrytis cinerea* is a major problematic issue for cultivated strawberries. Despite the application of fungicides, BFR can cause severe reduction (more than 50%) in commercial strawberry yields [7, 8]. *B. cinerea* infects both flowers and fruits, and it is often difficult to control under favorable environmental conditions. The development of BFR on strawberry fruit is a primary reason for fruit rejection by growers and consumers alike [6]. While the management of BFR typically relies on repeated applications of various groups of fungicides, the efficacy of these chemical treatments has significantly decreased due to the occurrence of fungicide-resistant isolates. Consequently, alternatives such as developing *B. cinerea* resistant cultivars are in high demand to mitigate the economic losses caused by BFR disease.

Several studies have been conducted to improve resistance against the devastating *B. cinerea* in cultivated strawberries [9–11]. The β–glucosidases (BGs), which play crucial roles in plant development and pathogen defense, are known to regulate abscisic acid (ABA) homeostasis and fruit ripening-related gene expression, and thus are significant players in BFR resistance [11]. In *FaBG3*-RNAi fruit, ABA content and gene expression related to cell wall catabolism (*FaPG1*, *FaEXP4*, *FaXYL1*, and *FaGAL1*) were substantially decreased, resulting in enhanced resistance against *B*. *cinerea*. A two-pore K^+^ channel gene (*FaTPK1*) regulating fruit ripening has been identified as a *Botrytis*-susceptible gene [10]. Resistance against *Botrytis* pathogen was found to decrease in *FaTPK1*-overexpressing fruit, while it increased in *FaTPK1*-RNAi fruit. It was also suggested that *FaWRKY11* is a positive regulator of plant resistance against *B. cinerea,* controlling genes related to phytohormone metabolism, including ABA and jasmonic acid (JA), and disease-resistance transcription factors (TFs) [9]. Resistance against *B. cinerea* was significantly increased in *FaWRKY11*-overexpressing fruit, while it slightly decreased in *FaWRKY11*-RNAi fruit.

*Arabidopsis* T-DNA knockout mutant lines have been widely utilized to study the gene functions for plant disease resistance [12, 13]. Screening of *Arabidopsis* mutants and identifying candidate genes for *B. cinerea* resistance can provide valuable insights into the mechanisms of BFR resistance in strawberries. The candidate genes identified in *Arabidopsis* can be tested for gene functions in octoploid strawberries by transient overexpression and RNA interference knockdown assays. Several genes have been reported for the resistance against *B. cinerea* in *Arabidopsis*. T-DNA insertion *Atmyb46* mutant lines exhibited enhanced resistance against *B*. *cinerea* in *Arabidopsis* leaves and transcriptional reprogramming of genes related to cell wall proteins and enzymes, including extracellular type III peroxidases [14]. An ethyl methanesulfonate-derived *Atocp3* mutant line also showed enhanced resistance against *B*. *cinerea* in *Arabidopsis* leaves, along with hydrogen peroxide (H_2_O_2_) accumulation and the transcriptional expression of marker genes, such as *Glutathione S-transferase1* and *Plant Defensin 1.2* (*PDF1.2*) [15]. Furthermore, CRISPR/Cas9-derived *VvWRKY52* (an ortholog gene of *AtWRKY53*) knockout mutants showed enhanced resistance against *B*. *cinerea* in grape leaves [16].

Unripe fruits infected with* Botrytis cinerea* usually lead to quiescent infections, while *B. cinerea* aggressively infects ripened fruits [17]. Plant hormones play a variety of roles in both strawberry fruit ripening and plant defense responses against *B. cinerea*. ABA is an inducer of fruit ripening, with ABA levels increasing gradually while auxin levels decrease gradually during fruit ripening. ABA signaling is involved in reactive oxygen species (ROS) production after *B. cinerea* infection [18–21]. The highest JA content is found in big green fruits, which drastically decreases in white fruits. JA and ET signaling are involved in plant defenses against necrotrophic pathogens including *B. cinerea*. External application of JA to red fruits has been shown to improve resistance against *B. cinerea* [5, 22]. Cell wall serves as a mechanical barrier against *B. cinerea* infection, and fruit ripening is associated with cell wall depolymerization and cell wall solubilization, leading to fruit softening that can facilitate *B. cinerea* infection [23]. Cell wall degradation not only makes it easier for *B. cinerea* to invade fruit cells but it also increases the content of fruit sugar, which *B. cinerea* utilizes [6]. Changes in secondary metabolites such as phenylpropanoids, flavonoids, hydrolysable tannins, and benzoic acids occur during fruit ripening and can also affect resistance against *B. cinerea* [24, 25].

*WRKY* transcription factors (TFs) play a significant role in regulating plant developmental processes, particularly in biotic and abiotic stress responses in plants, including *Arabidopsis*, rice, and tomato [26, 27]. *WRKY* TFs have been reported as modulators of plant immune responses against various pathogens, including *B. cinerea* [28–30]. *Camellia oleifera WRKY78* was significantly low in expression in anthracnose-resistant cultivars. Reduced anthracnose resistance was observed in *CoWRKY78* overexpression tobacco transgenic plants, exhibiting lower superoxide dismutase and peroxidase activities [28]. The promoter region of *Morus indica WRKY53* is induced by salicylic acid (SA). Overexpression of *MiWRKY53* in *Arabidopsis* altered not only leaf morphology, but also resistance against *Pseudomonas syringae* PstDC3000 [29]. *AtWRKY70* is associated with the regulation of the mutually antagonistic cross-talk between SA- and JA-dependent plant defense mechanisms. *AtWRKY70* expression is activated by external SA treatment and plays a role as an activator of SA-inducible genes but is repressed by external methyl jasmonate (MeJA) treatment and as a repressor of JA-inducible genes. After inoculation of the necrotrophic fungal pathogen, *Alternaria brassicicola*, the transcripts of the JA-responsive marker *PDF1.2* were highly accumulated in the *Atwrky70* mutant [31, 32]. *AtWRKY53* expression is strongly correlated with *AtWRKY70* expression after systemic acquired resistance induced by SA with the transcription cofactor *NPR1*. Both *AtWRKY53* and *AtWRKY70* have partially overlapping roles as positive regulators of plant basal defense responses against virulent *Pseudomonas syringae* [33]. *AtWRKY41* expression is immediately activated with *AtWKRY53* by flagellin treatment derived from *P. syringae*. Overexpression of *AtWRKY41* increases resistance to the hemi-biotrophic pathogen *P. syringae* but decreases resistance to the necrotrophic pathogen *Erwinia carotovora* [34]. *AtWRKY33* is a critical transcriptional regulator of plant immune responses, upregulated at early stages of *B. cinerea* infection in *Arabidopsis* [30, 35, 36]. Loss of function of *AtWRKY33* resulted in increased susceptibility to *B. cinerea*, while overexpression of the gene decreased susceptibility. *AtWRKY33* was found to regulate hormone signaling, redox homeostasis, and camalexin biosynthesis induced by the pathogen. *AtWRKY33* also affected the upstream regulation of *NCED3/NCED5*, negatively regulating ABA biosynthesis. Disruption of *AtWRKY33* function resulted in accumulation of ABA, which interacts with other plant defense hormones, such as jasmonic acid/ethylene (JA/ET) and SA. The antagonistic effect of ABA on JA was found to be a key regulator of resistance against *B. cinerea*, which is controlled by *AtWRKY33*. Additionally, single and double mutants of *AtWRKY3* and *AtWRKY4* were found to exhibit enhanced *B. cinerea* susceptibility, while AtWRKY8 was found to physically interact with AtVQ10 [37].

In addition to *Arabidopsis*, *WRKY* TFs in strawberries have been found to regulate plant defense responses to *B. cinerea* [5, 9]. It has been reported that *FaWRKY11* is involved in enhancing the resistance against *B. cinerea* in octoploid strawberry fruits [9]. Transient overexpression of *FaWRKY11* increased *B. cinerea* resistance in fruit, whereas transient knockdown of *FaWRKY11* decreased resistance against *B. cinerea*. Following *B. cinerea* infection, *FaJAZ1*, *FaJAZ4*, and *FaMAPK19* were highly expressed in *FaWRKY11*-overexpressing fruits. On the contrary, *FaWRKY25*, classified as group I *WRKY*, was found to be a susceptibility gene to *B. cinerea* in octoploid strawberries [5]. Transient overexpression of *FaWRKY25* increased *B. cinerea* susceptibility in strawberry fruits, while transient knockdown of *FaWRKY25* enhanced *B. cinerea* resistance. *FaWRKY25* expression gradually increased after *B. cinerea* infection. External JA application to fruits resulted in a decrease in *FaWRKY25* expression, leading to enhanced *B. cinerea* resistance. Genes related to JA biosynthesis and metabolism, such as *FaLOX*, *FaAOS*, *FaAOC*, and *FaOPR3*, were highly expressed in *FaWRKY25* knockdown fruits during fruit maturation. Following *B. cinerea* infection, *FaCOI1*, *FaMYC2*, and *FaJAZ12* were highly expressed in *FaWRKY25* knockdown fruits compared to mock fruits.

In this study, we identified and investigated the role of two *WRKY* genes, *FaWRKY29* and *FaWRKY64*, both of which belong to the strawberry *WRKY* group III, in conferring resistance to BFR in cultivated strawberries. Recently, 64 *WRKY* TFs of *Fragaria vesca* and 257 *WRKY* TFs of *Fragaria* × *ananassa* (cv. ‘Camarosa’) were renamed by Garrido-Gala et al. [38], respectively, and we followed the nomenclature shown in the publication. Transient knockdown experiments on each of two genes, *FaWRKY29* and *FaWRKY64*, were performed to determine their effect on *B*. *cinerea* resistance in strawberry fruits, and analyzed transcriptome data to identify differentially expressed genes. Our findings indicate that *FaWRKY29* and *FaWRKY64* genes are *B*. *cinerea* susceptible genes for developing *B*. *cinerea* resistant strawberries through CRISPR/Cas9-mediated mutagenesis.

## Results

### Identification of candidate genes for *Botrytis cinerea* resistance in octoploid strawberry

**Fig. 1:**
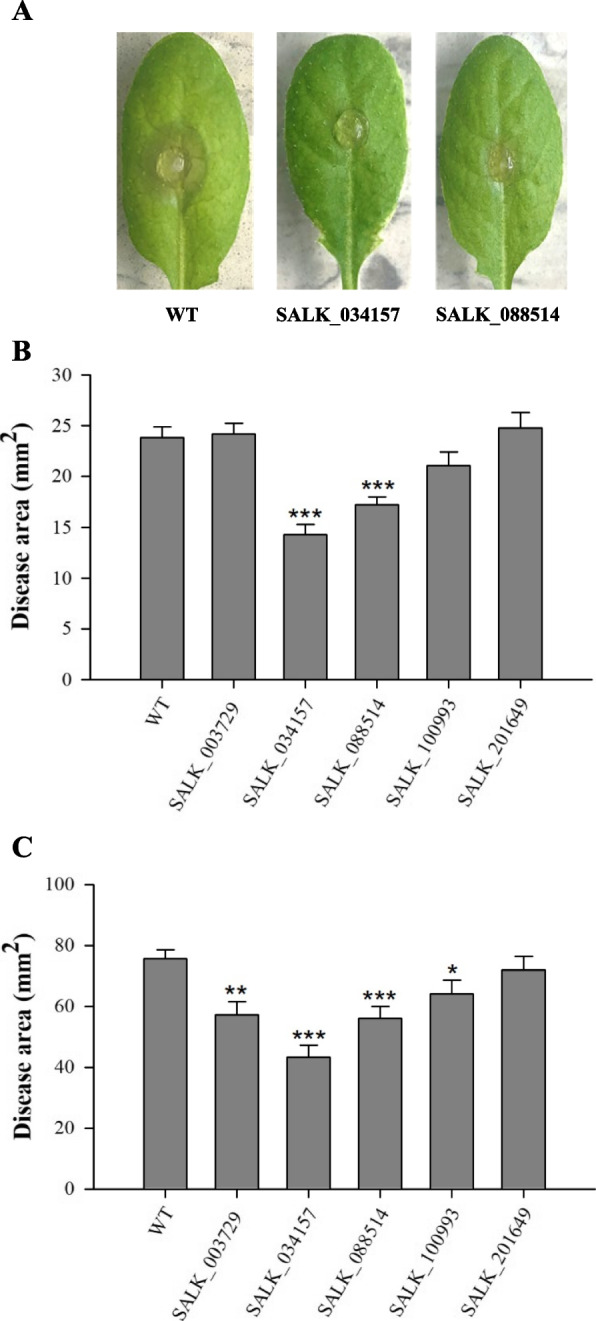
*Botrytis cinerea* inoculation to *Arabidopsis* T-DNA knockout lines. *Botrytis cinerea* was inoculated to 19 – 24 leaves from 4-week-old *Arabidopsis* plants for each homozygous mutant line. **A** *Botrytis cinerea* inoculated leaf pictures were taken one day after inoculation from wild type (WT), SALK_034157, and SALK_088514. The disease area was measured two days after inoculation (**B**) and three days (**C**) using the ImageJ program. Col-0 was used as WT. The experiment was repeated two times. Error bars indicate standard error. Asterisks indicate significant differences from the WT determined by the Student’s t-test (* *p* < 0.05, ** *p* < 0.01, and *** *p* < 0.001)

**Fig. 2:**
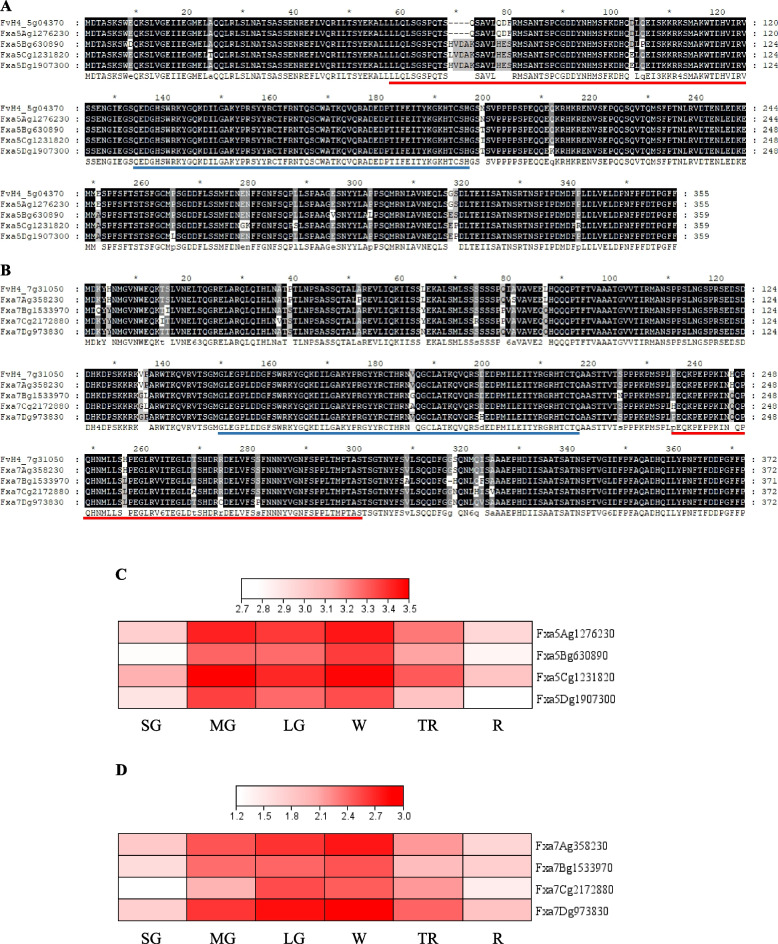
Multiple alignments of amino acid sequences for *WRKY29* and *WKRY64* in *Fragaria vesca* and *Fragaria* × *ananassa* (cv. ‘Florida Brilliance’). **A** *AtWRKY53* (At4g23810), *FvWRKY29* (FvH4_5g04370), and *FaWRKY29* homoeologs. **B** *AtWRKY53*, *FvWRKY64* (FvH4_7g31050), and *FaWRKY64* homoeologs. Red bars indicate RNAi targeting regions. Blue bars indicate WRKY domains. Expression profile of homoeologous copies of *FaWRKY29* (**C**) and *FaWRKY64* (**D**) in six different fruit developmental stages of ‘Florida Brilliance’; Small Green (SG), Medium Green (MG), Large Green (LG), White (W), Turning Red (TR), and Red (R). The gene expression levels were represented as log2-transformed depth-normalized counts (CPM+1). To visualize the gene expression profile, a heatmap was generated using TBtools

From a search of public database and previous publications, we identified several candidate genes, *AtDND1* (At5g15410) [39], *AtMPK3* (At3g45640) [40], *AtOCP3* (At5g11270) [15], *AtMYB46* (At5G12870) [14], *VvWRKY52* (*AtWRKY53*, At4g23810) [16]. These genes have been reported to be involved in plant defense responses against BFR in plants. *Arabidopsis* mutants obtained from the *Arabidopsis* Information Resource (TAIR, https://www.arabidopsis.org) were inoculated with strawberry field isolates of *B. cinerea* (Table S1). In this study, we identified three candidate genes, *Arabidopsis OVEREXPRESSOR OF CATIONIC PEROXIDASE 3* (*OCP3*), *MYB46*, and *WRKY53*, potentially associated with the resistance of BFR in strawberry. T-DNA inserted *Atmyb46* mutants and ethyl methanesulfonate-derived *Atocp3* mutants exhibited enhanced *B. cinerea* resistance in *Arabidopsis* [14, 15]. CRISPR/Cas9-derived *VvWRKY52* (an ortholog gene of *AtWRKY53*) knockout mutants showed enhanced *B. cinerea* resistance in grape [16]. Homozygous seeds of T-DNA insertion mutant lines for each of the three genes were obtained from TAIR. The homozygous plants were confirmed by PCR (Fig. S1). After *B. cinerea* inoculation, necrosis symptoms in *Arabidopsis* leaves were notably less evident in mutants of *AtWRKY53* (SALK_034157), *AtMYB46* (SALK_088514), and *AtOCP3* (SALK_003729) compared to wild type (WT) (Fig. 1A). Two days after *B. cinerea* inoculation, the disease area was significantly reduced in SALK_034157 and SALK_088514 compared to WT, with the smallest area observed in SALK_034157 (Fig. 1B). Three days after *B. cinerea* inoculation, the disease area was significantly reduced in SALK_003729, SALK_034157, SALK_088514, and SALK_100993 compared to WT, with the smallest area also observed in SALK_034157 (Fig. 1C). The SALK_034157 mutant has T-DNA insertion on *AtWRKY53*, and the ortholog of this gene was used for further validation of resistance against *B. cinerea* in strawberry fruits. With the amino acid sequence of *AtWRKY53*, we identified two ortholog genes, *FaWRKY29* on chromosome 5 and *FaWRKY64* on chromosome 7, both of which are classified into *WRKY* group III. The gene names of *FaWRKY29* and *FaWRKY64* were followed by the nomenclature of strawberry *WRKY* TFs described by Garrido-Gala et al. [38]. The cDNA sequences of *FaWRKY29* or *FaWRKY64* were used to identify their homoeologous sequence in the telomere-to-telomere quality reference genome of octoploid strawberry, ‘Florida Brilliance’ (FaFB1) [4]. In total, we identified four homoeologs both for *FaWRKY29* and *FaWRKY64* in the reference genome of FaFB1, respectively (Fig. 2A and B). Using our previous transcriptome data available from ‘Florida Brilliance’, the expression patterns of *FaWRKY29* and *FaWRKY64* were determined in six different stages of fruit development (Small Green, SG; Medium Green, MG; Large Green, LG; White, W; Turning Red, TR; and Red, R) (Fig. 2C and D). It was found that all homoeologous copies of the *FaWRKY29* and *FaWRKY64* were constitutively expressed in the main stages of fruit development – MG, LG, W, and TR. Two homoeologous copies of *FaWRKY29* (Fxa5Ag1276230 and Fxa5Cg1231820) and three for *FaWRKY64* (Fxa7Ag358230, Fxa7Bg1533970, and Fxa7Dg973830) were dominantly expressed in all fruit development stages. All copies of *FaWRKY29* and *FaWRKY64* showed their highest expression levels at stage W (fruit stage used for *Agrobacterium* infiltration for RNAi gene silencing) and slightly decreased at stage TR. RNAi constructs targeting all the homoeologs of *FaWRKY29* or *FaWRKY64* were specifically designed, avoiding the *WRKY* conserved domain.

### Enhanced resistance against *B. cinerea* in strawberry fruits with knockdown of *FaWRKY29* or *FaWRKY64*

**Fig. 3:**
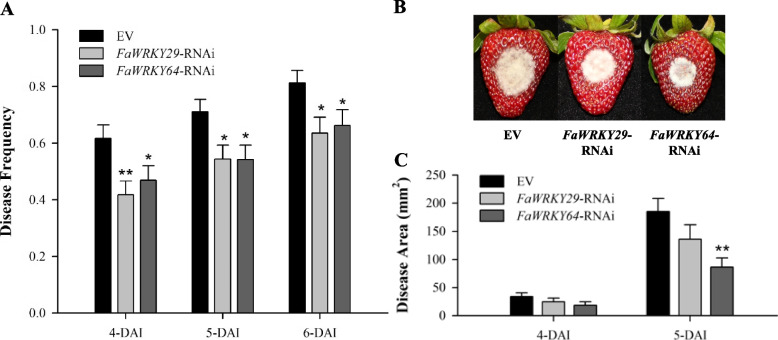
*Botrytis cinerea* inoculation assay on *Agrobacterium* infiltrated strawberry fruits. **A** Red strawberry fruits were infected with *Botrytis cinerea* five days after *Agrobacterium* infiltration. Disease frequency was calculated four, five, and six days after inoculation (DAI) with *Botrytis cinerea*. **B** Disease symptoms on strawberry fruits were taken pictures at 5-DAI. **C** Disease area was measured using the ImageJ program at 4- and 5-DAI. The experiment was repeated four times. Error bars indicate standard error. Asterisks indicate significant differences from the EV at zero days determined by the Student’s t-test (*p < 0.05, ** *p* < 0.01)

A *Botrytis cinerea* infection assay was performed to examine whether transient knockdown of *FaWRKY29* or *FaWRKY64* could enhance resistance in strawberry fruits. White stage fruits were infiltrated with *Agrobacterium* harboring the RNAi vectors (*FaWRKY29*-RNAi or *FaWRKY64*-RNAi) or an empty vector (EV). Five days after *Agrobacterium* infiltration (0 days after *B. cinerea* inoculation), the expression of *FaWRKY29* and *FaWRKY64* was significantly decreased in *FaWRKY29*-RNAi and *FaWRKY64*-RNAi fruits compared to the EV (Fig. S2A and B). Four days after *B. cinerea* inoculation, the expression levels of *FaWRKY29* and *FaWRKY64* were generally reduced in EV fruits and the corresponding RNAi fruits compared to those of 0 days after *B. cinerea* inoculation. Disease frequency was calculated by dividing the number of BFR developed fruits by the number of *B. cinerea* inoculated fruits. Five days after *B. cinerea* infection in fruits, disease frequencies were significantly decreased in both *FaWRKY29* and *FaWRKY64* knockdown fruits compared to fruits containing the EV (Fig. 3A). Delayed disease occurrence was observed in fruits with transiently knockdown of *FaWRKY29* or *FaWRKY64* at all time points. Five days after inoculation (DAI) of *B. cinerea*, strawberry fruits were severely infected with *B. cinerea*, and mycelia were visible on the surface of the fruits treated with each RNAi or the EV (Fig. 3B). A significant reduction in disease area was observed in *FaWRKY64* knockdown fruits compared to the EV at 5-DAI, but not in *FaWRKY29* knockdown fruits (Fig. 3C). Taken together with the *B. cinerea* inoculation results, transient knockdown of *FaWRKY29* or *FaWRKY64* led to decreased disease symptoms and reduced growth of *B. cinerea* in the infected fruits.

### Differentially expressed genes in response to *B. cinerea* in strawberry fruits with knockdown of *FaWRKY29* or *FaWRKY64*

Five days after agroinfiltration, *FaWRKY29*-RNAi, *FaWRKY64*-RNAi, EV, or mock agroinfiltrated fruits were inoculated with *B. cinerea*. For transcriptome data analysis, fruit samples were collected 0-DAI or 4-DAI. RNA sequencing data were mapped to the octoploid strawberry ‘Camarosa’ reference genome [2]. Approximately 91.4% of RNA reads were successfully mapped to the reference genome (Table S2, NCBI BioProject number: PRJNA946145). Differentially expressed genes (DEGs) were determined by comparing normalized transcript abundances using a threshold (≥ threefold with *p*-value ≤ 0.05) between the RNA-seq samples of EV vs. mock control, *FaWRKY29-*RNAi vs. EV, and *FaWRKY64-*RNAi vs. EV at 0-DAI and 4-DAI of *B. cinerea* (Table S3). The term DEG indicates a gene with different expression levels, exhibiting a fold change of three or more between the compared samples. We applied a fold change of three or more to emphasize genes that exhibit drastic expression change rather than a fold change of two or more.

**Fig. 4:**
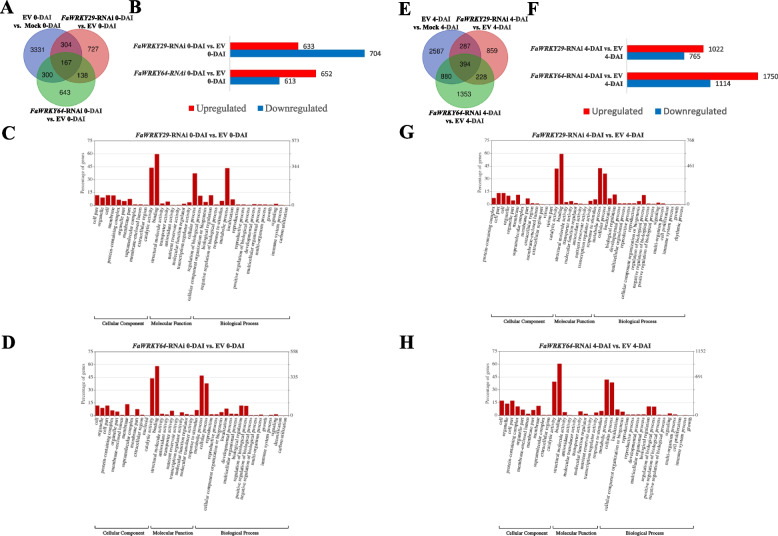
Differentially expressed genes (DEGs) and GO terms in *Botrytis cinerea* infected strawberry fruits. Venn diagram depiction among three pairwise libraries including EV vs. Mock, *FaWRKY29*-RNAi vs. EV, and *FaWRKY64*-RNAi vs. EV at 0-DAI (**A**) and 4-DAI (**E**). Histogram representing the number of DEGs detected on pairwise comparison zero (**B**) and four (**F**) days after *B. cinerea* infection. GO terms assignment for the strawberry transcriptome on pairwise *FaWRKY29*-RNAi 0-DAI vs. EV 0-DAI (**C**), *FaWRKY64*-RNAi 0-DAI vs. EV 0-DAI (**D**), *FaWRKY29*-RNAi 4-DAI vs. EV 4-DAI (**G**), and *FaWRKY64*-RNAi 4-DAI vs. EV 4-DAI (**H**). Results are summarized into three main GO categories of cellular component, molecular function and biological process. The left y-axis represents the percentage of specific category of genes present in each main category whereas, the right y-axis indicates the gene number in the same category

At 0-DAI, a total of 1,337 (633 downregulated and 704 upregulated) in *FaWRKY29*-RNAi and 1,265 genes (652 downregulated and 613 upregulated) in *FaWRKY64*-RNAi were differentially expressed compared to the EV (Fig. 4A and B). In addition, 305 genes were identified in both *FaWRKY29*-RNAi and *FaWRKY64*-RNAi compared to the EV. At 4-DAI, a total of 1,787 (1,022 downregulated and 765 upregulated) in *FaWRKY29*-RNAi and 2,864 genes (1,750 downregulated and 1,114 upregulated) in *FaWRKY64*-RNAi were differentially expressed compared to the EV (Fig. 4E and F). In addition, 622 genes were simultaneously identified in *FaWRKY29*-RNAi and *FaWRKY64*-RNAi compared to the EV.

Global analyses of *FaWRKY29* or *FaWRKY64* knockdown fruits DEGs were performed to provide an overview of gene expression before and after *B. cinerea* inoculation. Enrichment of gene ontology terms was analyzed, and DEGs were classified by cellular components, molecular function, and biological process (Fig. 4). As a transcription factor, knockdown of *FaWRKY29* or *FaWRKY64* principally altered gene expressions related to ‘binding’ and ‘catalytic activity’. In molecular function, ‘binding’ and ‘catalytic activity’ were the two most enriched groups. At 0-DAI, 455 (66.72%) DEGs and 433 (66.11%) DEGs were grouped into ‘binding’ in *FaWRKY29-*RNAi fruit and *FaWRKY64-*RNAi fruit, respectively (Fig. 4C and D). At 0-DAI, 337 (49.41%) DEGs and 328 (50.08%) DEGs were grouped into ‘catalytic activity’ in *FaWRKY29*-RNAi fruit and *FaWRKY64*-RNAi fruit, respectively. The ratios of DEGs grouped into ‘binding’ at 4-DAI were similar to those of 0-DAI, but the numbers of DEGs were increased at 4-DAI compared to 0-DAI (Fig. 4G and H).

**Fig. 5:**
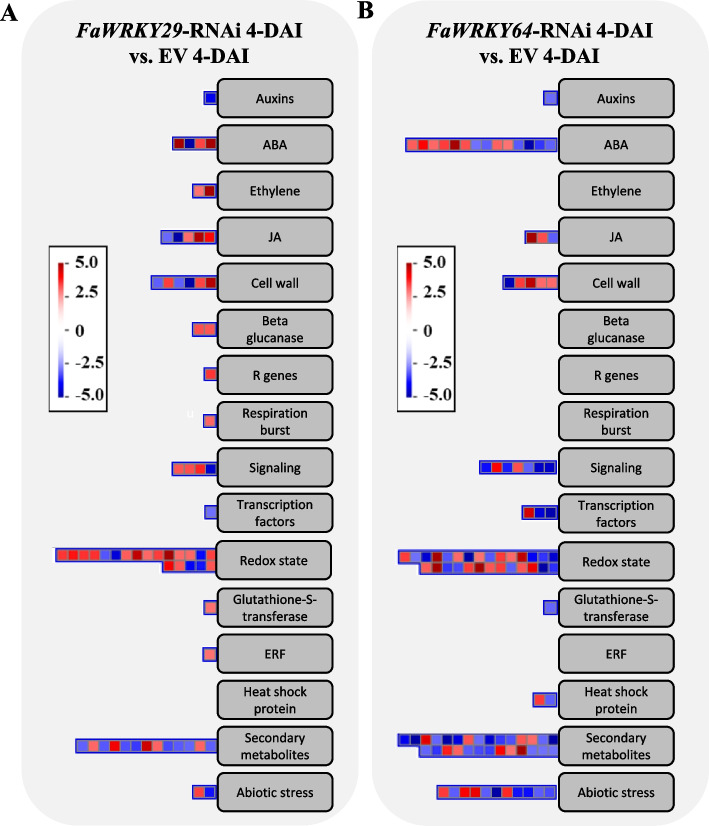
MapMan overview map at four days after inoculation (DAI). Differentially expressed genes specific to *FaWRKY29-*RNAi fruits (**A**) and *FaWRKY64-*RNAi fruits (**B**). Red: upregulation and Blue: downregulation of genes

Mapman analyses were performed to characterize the biological relevance of DEGs resulting from *FaWRKYs* knockdown and *B. cinerea* infection (Fig. 5 and Fig. S3). The *FaWRKY29-*RNAi-specific DEGs (727 in Fig. 4A and 859 in Fig. 4E), the *FaWRKY64*-RNAi-specific DEGs (643 in Fig. 4A and 1,353 in Fig. 4E) were classified in the ‘biotic stress’ through Mapman analyses. Within the ‘biotic stress’ category, most of the DEGs classified in ‘Redox state’, ‘Secondary metabolites’, ‘Cell wall’, and ‘ABA’ for both *FaWRKY29*-RNAi DEGs and *FaWRKY64*-RNAi DEGs at 0-DAI (Fig. S3). DEGs involved in ‘JA’ were observed in both *FaWRKY29*-RNAi DEGs and *FaWRKY64*-RNAi DEGs at 0-DAI, but those related to ‘SA’ were not observed. DEG involved in ‘Ethylene’ was observed in *FaWRKY29*-RNAi DEGs, but not in *FaWRKY64*-RNAi DEGs. At 4-DAI, DEGs classified in the ‘biotic stress’ category showed similar distribution patterns to those at 0-DAI for both *FaWRKY29-*RNAi DEGs and *FaWRKY64*-RNAi DEGs (Fig. 5). Most DEGs were classified under ‘Redox state’, ‘Secondary metabolites’, ‘Cell wall’, and ‘ABA’ for both *FaWRKY29*-RNAi DEGs and *FaWRKY64*-RNAi DEGs at 4-DAI. DEGs related to ‘JA’ were observed in both *FaWRKY29*-RNAi DEGs and *FaWRKY64*-RNAi DEGs at 4-DAI, but those related to ‘SA’ were not observed. DEGs involved in ‘Ethylene’ were observed in *FaWRKY29*-RNAi DEGs but not in *FaWRKY64*-RNAi DEGs. There were more DEGs in the ‘biotic stress’ category for *FaWRKY29*-RNAi DEGs and *FaWRKY64*-RNAi DEGs at 4-DAI than at 0-DAI, respectively. More DEGs were observed in ‘Signaling’ for both *FaWRKY29*-RNAi DEGs and *FaWRKY64*-RNAi DEGs at 4-DAI than at 0-DAI. A greater number of DEGs were observed in the ‘Abiotic stress’ category for *FaWRKY64*-RNAi DEGs at 4-DAI compared to 0-DAI.

Following *B. cinerea* infection (4-DAI), numerous DEGs involved in plant cell wall and ROS were identified in the ‘Redox state’, ‘Secondary metabolites’, and ‘Cell Wall’ categories (Table 1) [41–48]. Genes regulating cell wall composition and stiffening were identified in *FaWRKY29*-RNAi DEGs and/or *FaWRKY64*-RNAi DEGs in response to *B. cinerea*. Two *pectin methylesterase* (*PME,* Fxa1Dg717060, and Fxa7Ag354550) genes were identified in *FaWRKY64*-RNAi-specific DEGs and six *PME* (Fxa4Cg522800, Fxa4Cg522810, Fxa6Bg1778560, Fxa7Ag336960, Fxa7Ag714250, and Fxa7Cg2153620) genes were identified in *FaWRKY29*-RNAi-specific DEGs. Fxa7Ag354550 in *FaWRKY64-*RNAi-specific DEGs and Fxa7Cg2153620 and Fxa7Ag336960 in *FaWRKY29* are functionally associated with the resistance against *B. cinerea* [41]. *Cytochrome P450-dependent fatty acyl omega-hydroxylase* (Fxa5Ag1274450, Fxa5Cg1230200, and Fxa5Cg1230210) genes were identified in *FaWRKY29*-RNAi-specific DEGs. In addition, genes that regulate ROS production and degradation were identified in *FaWRKY29*-RNAi DEGs and/or *FaWRKY64*-RNAi DEGs (Table 1). *NADPH-oxidase* (*Rboh*, Fxa5Cg1230750), *M-type thioredoxin* (Fxa6Dg750700), and *atypical 2-Cys peroxiredoxin* (*PrxQ*, Fxa1Bg2233150) genes were identified in *FaWRKY64*-RNAi-specific DEGs. *H-type thioredoxin* (Fxa6Cg2410140), *O-type thioredoxin* (Fxa3Dg2298420), mRNA-binding regulatory factor (TZF, Fxa1Ag149970), and *PrxQs* (Fxa1Cg1501580 and Fxa1Dg714640) genes were identified in *FaWRKY29*-RNAi-specific DEGs. *H-type thioredoxin* (Fxa2Bg966150) and *M-type thioredoxin* (Fxa6Cg2434130) genes were identified in both *FaWRKY29-* and *FaWRKY64*-RNAi DEGs.

**Table 1:** Differentially expressed genes related to cell wall composition and reactive oxygen species in response to *B. cinerea* in *FaWRKY29* or *FaWRKY64* knockdown strawberry fruits after *B. cinerea* infection

Gene ID (‘Camarosa’)	Gene ID (‘Florida Brilliance’)	Gene	Gene Functions	Reference
maker-fvb5-4-snap-gene-218.34-mrna-1	Fxa1Dg717060	Pectin methylesterase	Plant invertase/pectin methylesterase inhibitor superfamily	[41, 42]
maker-fvb4-2-snap-gene-91.29-mrna-1	Fxa4Cg522800 Fxa4Cg522810	Pectin methylesterase	Plant invertase/pectin methylesterase inhibitor superfamily	
maker-fvb6-3-augustus-gene-104.46-mrna-1	Fxa6Bg1778560	Pectin methylesterase	Pectin methylesterase 3	
maker-fvb7-2-augustus-gene-99.50-mrna-1	Fxa7Ag336960	Pectin methylesterase	Plant invertase/pectin methylesterase inhibitor superfamily	
maker-fvb7-1-augustus-gene-277.59-mrna-1	Fxa7Ag354550	Pectin methylesterase	Plant invertase/pectin methylesterase inhibitor superfamily	
maker-fvb1-1-augustus-gene-230.34-mrna-1	Fxa7Ag714250	Pectin methylesterase	Pectin methylesterase 44	
maker-fvb7-1-snap-gene-152.49-mrna-1	Fxa7Cg2153620	Pectin methylesterase	Plant invertase/pectin methylesterase inhibitor superfamily	
snap_masked-fvb5-1-processed-gene-14.34-mrna-1	Fxa5Ag1274450	Fatty acyl omega-hydroxylase	Cytochrome P450	[43]
maker-fvb5-4-snap-gene-14.60-mrna-1	Fxa5Cg1230200 Fxa5Cg1230210	Fatty acyl omega-hydroxylase	Cytochrome P450	
maker-fvb2-4-augustus-gene-157.55-mrna-1	Fxa2Bg948330 Fxa2Bg948340	Catalytic component CesA of cellulose synthase complex	Cellulose synthase A4	[44]
maker-fvb5-4-augustus-gene-16.48-mrna-1	Fxa5Cg1230750	NADPH-oxidase (Rboh)	Respiratory burst oxidase protein F	[45]
augustus_masked-fvb1-4-processed-gene-28.4-mrna-1	Fxa1Ag149970	mRNA-binding regulatory factor (TZF)	CCCH-type zinc finger family protein	[46]
maker-fvb2-4-snap-gene-248.57-mrna-1	Fxa2Bg966150	H-type thioredoxin	β-galactosidase 5	[47]
snap_masked-fvb6-1-processed-gene-1.47-mrna-1	Fxa6Cg2410140	H-type thioredoxin	Thioredoxin 2	
maker-fvb6-2-augustus-gene-89.30-mrna-1	Fxa6Cg2434130	M-type thioredoxin	Thioredoxin superfamily protein	
maker-fvb6-4-augustus-gene-197.28-mrna-1	Fxa6Dg750700	M-type thioredoxin	Thioredoxin superfamily protein	
maker-fvb3-3-augustus-gene-55.22-mrna-1	Fxa3Dg2298420	O-type thioredoxin	Thioredoxin O2	
maker-fvb1-2-augustus-gene-64.50-mrna-1	Fxa1Bg2233150	Atypical 2-Cys peroxiredoxin	Thioredoxin superfamily protein	[48]
maker-fvb1-3-augustus-gene-44.33-mrna-1	Fxa1Cg1501580	Atypical 2-Cys peroxiredoxin	Thioredoxin superfamily protein	
maker-fvb1-1-augustus-gene-232.33-mrna-1	Fxa1Dg714640	Atypical 2-Cys peroxiredoxin	Thioredoxin superfamily protein	

## Discussion

*Botrytis cinerea* has a wide range of host plants, and the pathogen causing BFR in strawberry fruits can also cause disease symptoms in *Arabidopsis* [49]. In this study, we investigated candidate genes for *Botrytis* resistance in public databases and screened *Arabidopsis* mutant lines with strawberry field isolates of *B. cinerea*. As shown in Fig. 1, the *Atwrky53* mutant showed enhanced disease resistance at 1-DAI. The disease area was the smallest in the *Atwrky53* mutant at both 2-DAI and 3-DAI. It has been known that most of the group III *Arabidopsis* *WRKY* TFs participate in plant-pathogen interactions [50]. *AtWRKY53* plays an important functional role in the senescence regulatory network, connecting with plant hormones such as SA and JA, ROS, and other TFs [51]. It has also been reported that *AtWRKY53* is highly upregulated by ROS and associated with the regulation of the mutually antagonistic cross-talk between SA and JA in response to biotic stresses. Interestingly, a recent study showed that CRISPR/Cas9-derived *VvWRKY52* mutants showed increased resistance in *B. cinerea* infected leaves [16]. However, the mode of action for *VvWRKY52*-mediated resistance against *B. cinerea* in grapes remains unknown. Another study demonstrated that the expression of *VqWRKY52* was strongly activated by SA, and ectopic overexpression of *VqWRKY52* in *Arabidopsis* increased susceptibility to *B. cinerea* [52].

Our results showed that the knockdown of two strawberry orthologs of *AtWRKY53*, *FaWRKY29* or *FaWRKY64*, led to a decrease in disease frequency and disease area after *B. cinerea* inoculation and induced changes in the expression of multiple defense-related genes, indicating their potential role in the regulation of plant immunity against *B. cinerea* in strawberry. In this study, we performed Mapman analyses on DEGs to better understand the biological relevance of these genes and *B. cinerea* resistance (Fig. 5 and Fig. S3). Results showed that after *B. cinerea* infection, the plant stress hormones ABA and JA were over-represented in the DEGs from *FaWRKY29*-RNAi and *FaWRKY64*-RNAi. The plant stress hormones ABA, JA, SA, and ET play a crucial role in controlling plant defense mechanisms against pathogens [53]. JA and ET are typically involved in plant defense against necrotrophic pathogens such as *B. cinerea*, but the signaling pathways for these hormones can interact reciprocally with the SA signaling pathway [17]. Generally, ABA can repress plant resistance by antagonistically regulating the SA- and JA/ET-dependent defense pathways. However, ABA has also been reported to positively impact JA and SA signaling components [36]. Our results suggest that the knockdown of *FaWRKY29* and *FaWRKY64* primarily altered the expression of genes related to ABA and JA signaling, while genes related to ET and SA signaling might be regulated in an antagonistic manner by ABA and/or JA signaling.

A strawberry *mRNA-binding regulatory factor* (*TZF*, Fxa1Ag149970) gene, involved in JA signaling and ROS, was identified in *FaWRKY29*-RNAi-specific DEGs (Table 1). *AtOZF1* (an *Arabidopsis* ortholog) expression was highly induced by JA, and *AtOZF1* positively regulated the expression of genes in JA signaling such as *AtPDF1.2*, *AtVSP2*, *AtTHI2.1*, and *AtORA59* [54]. *AtOZF1* was involved in both NON-EXPRESSOR of PR1 (NPR1)-dependent and NPR1-independent SA-signaling. *AtOZF1* plays a role in SA-JA cross-talk like *AtNPR1*. Loss-of-function of *OZF1* enhanced susceptibility to *B. cinerea* in *Atozf1* mutants. After *B. cinerea* infection, the strawberry *FaTZF* was upregulated (fold change 4.57) in *FaWRKY29* knockdown fruits, which can reduce *B. cinerea* susceptibility in infected fruits.

Changes in the expression of plant hormone-related genes (ABA and JA) appear to affect plant cell wall composition and ROS production. Knockdown of *FaWRKY29* and *FaWRKY64* genes resulted in altered expression of genes associated with cell walls and ROS in *B. cinerea* infected fruits (Table 1). Plants defend against pathogens through a combination of mechanisms, including recognition, signaling, cell wall remodeling, and cell death. The cuticle and cell wall serve as the primary barriers to pathogens and are penetrated by fungal pathogens through physical or chemical means. Necrotrophic plant pathogens like *B. cinerea* attack plant cuticles and cell walls by releasing hydrolyzing enzymes such as cutinases, PMEs, and polygalacturonases [17, 55, 56].

PMEs are associated with pectin remodeling and disassembly of cell walls and play an important role in multiple biotic stresses like *B. cinerea* infection [41]. Several strawberry *PMEs* were identified in the DEGs related to cell wall composition in response to *B. cinerea* (Table 1). Three strawberry *PMEs* (Fxa7Ag336960, Fxa7Ag354550, and Fxa7Cg2153620) were orthologs of *AtPME17* (AT2G45220), which is highly expressed after *B. cinerea* infection [41]. ABA, JA, SA, and ET signaling networks were involved in *AtPME17* expression during *B. cinerea* infection. Lesion area was significantly increased in *B. cinerea* inoculated leaf tissues in *Atpme17* mutants, suggesting the role of *AtPME17* in plant defense signaling responses. Additionally, a significant decrease in *Plant Defensin 1.2* (*PDF1.2*, a marker for the JA/ET signaling pathways) expression was observed in *Atpme17* mutants. *AtPME17* highly contributes to *B. cinerea* resistance in *Arabidopsis* by activating JA/ET-dependent *PDF1.2* expression. In this study, three strawberry *PMEs* were upregulated by *B. cinerea* infection, which may reduce *B. cinerea* susceptibility in both *FaWRKY29* and *FaWRKY64* knockdown fruits by pectin remodeling.

ROS plays numerous important roles in plant physiology, development, and cellular signaling. In general, high ROS levels trigger detrimental effects such as lipid peroxidation in cellular membranes, DNA damage, and protein denaturation in plant cells. At the same time, ROS is an important signaling molecule and plays an essential role in response to various biotic stresses [57]. Pathogen attack by *B. cinerea* triggers plants to produce large amounts of ROS in the early plant-pathogen interaction, known as the oxidative burst. The oxidative burst can induce one of the plant defense mechanisms, hypersensitive cell death [58]. The major types of ROS are H_2_O_2_, superoxide (O_2_^−^), and hydroxyl radicals (OH•). Scavenging of extra ROS can be conducted by antioxidant enzymes such as superoxide dismutase, catalase, Prx, and ascorbate peroxidase [59]. Cell death caused by excessive ROS production promotes *B. cinerea* infection. Thus, the inhibition of ROS formation in infected tissue can improve resistance against *B. cinerea* [60]*.* Overexpression of *BnaWGR1*, an oilseed rape ortholog of *AtWRKY53*, increased ROS levels and induced cell death in *Arabidopsis* leaves [61].

Strawberry *NADPH-oxidase* (*Rboh*, Fxa5Cg1230750) gene related to ROS was identified in *FaWRKY64*-RNAi-specific DEGs (Table 1), and it was an ortholog of *AtRBOHF* (AT1G64060). *AtRBOHF* is necessary to accumulate ROS by an apoplastic oxidative burst in the plant defense responses [62]. In addition, *AtRBOHD* and *AtRBOHF* are involved in ROS-dependent ABA signaling, resulting in ABA-induced ROS production in guard cell regulation [20]. After *B. cinerea* infection, the strawberry *FaRboh* was downregulated (fold change -4.81) in *FaWRKY64* knockdown fruits. The downregulation of *FaRboh* can reduce ABA-induced ROS production in infected fruit, which can contribute to the enhancement of *B. cinerea* resistance in *FaWRKY64* knockdown fruits.

**Fig. 6:**
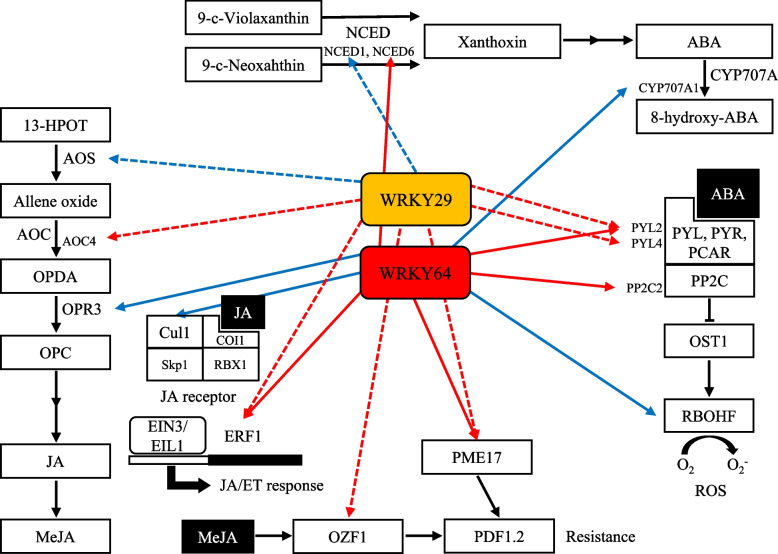
A hypothetical model representing the enhancement of resistance against *Botrytis cinerea* in the *FaWRKY29* or *FaWRKY64* knockdown strawberry fruits. Differentially expressed genes identified in *FaWRKY29* and/or *FaWRKY64* knockdown fruits in response to *B. cinerea* were represented. Dotted lines indicate *FaWRKY29*, while solid lines indicate *FaWRKY64*. The red color represents upregulation upon *FaWRKY29* or *FaWRKY64* knockdown, whereas the blue color signifies downregulation

Our transcriptome results showed that the enhanced resistance against *B. cinerea* in *FaWRKY29* and *FaWRKY64* knockdown fruits appears to be modulated by complex interactions between ABA signaling, JA signaling, and ROS accumulation. As shown in Fig. 6, we suggest a hypothetical model of the enhancement of *B. cinerea* resistance in *FaWRKY29* or *FaWRKY64* knockdown fruits in response to *B. cinerea* based on our transcriptome analysis. The knockdown of *FaWRKY29* or *FaWRKY64* leads to the regulation of genes involved in both JA biosynthesis (*AOS*, *AOC4*, and *OPR3*) and JA signaling (*Cul1* and *ERF1*). Additionally, it triggers the activation of *PME17* and *OZF1* gene expression, which positively regulate JA/ET‐dependent *PDF1.2* expression. Consequently, *FaWRKY29* or *FaWRKY64* knockdown plays a significant role in enhancing *B. cinerea* resistance through the JA/ET plant defense pathways. Furthermore, the knockdown of *FaWRKY29* or *FaWRKY64* also influences the expression of genes associated with ABA biosynthesis (*NCED1*, *NCED4*, and *CYP707A1*), along with ABA signaling genes (*PYL2*, *PYL4*, and *PP2C2*). Also, it negatively regulates *RBOHF* expression, involved in ABA-induced ROS production. The reduction of ROS in infected fruit could contribute to enhanced *B. cinerea* resistance.

A low level of ABA can enhance *B. cinerea* resistance and induce early and strong H_2_O_2_ accumulation in *B. cinerea* infected tissues [63]. After *B. cinerea* infection, strawberry genes that might be involved in pectin and cuticle development (such as *PMEs* and *fatty acyl omega-hydroxylases*) were modulated by the knockdown of *FaWRKY29* and/or *FaWRKY64*. Since it is known that *AtPME17*, *AtT1*, and *AtLCR* have critical roles in proper pectin and cuticle development [64–66]. Moreover, upregulation of *AtPME17* and *AtOZF1* could enhance the resistance against *B. cinerea*, as the genes positively regulate JA/ET‐dependent *PDF1.2* expression [41, 54]. Strawberry genes involved in ROS production and ROS scavenging (such as *FaRboh*, *FaTZF*, *thioredoxins*, and *peroxiredoxins*) were also regulated by knockdown of *FaWRKY29* and/or *FaWRKY64*. *FaWRKY64* knockdown fruits showed relatively stronger *B. cinerea* resistance than *FaWRKY29* knockdown fruits. The total number of DEGs classified in ‘Biotic stress’ was higher in *FaWRKY64*-RNAi-specific DEGs than in *FaWRKY29*-RNAi-specific DEGs (Fig. 5).

## Conclusion

In this study, we employed a reverse genetics approach to screen *Arabidopsis* T-DNA knockout mutants reported in the plant defense pathway against *B. cinerea*. The mutants for *AtWRKY53* showed enhanced resistance in response to *B. cinerea* infection. Two orthologs of this gene, *FaWRKY29* and *FaWRKY64*, were identified in octoploid strawberries, and transient knockdown of either *FaWRKY29* or *FaWRKY64* led to enhanced resistance against *B. cinerea* in fruits. Genes involved in ABA and JA signaling, as well as genes related to plant cell wall composition, decomposition (*PME* and *fatty acyl omega-hydroxylases*), and ROS (*Rboh* and *PrxQ*), were over-represented in *FaWRKY29-* and *FaWRKY64*-RNAi DEGs after *B. cinerea* infection. This suggests that the knockdown of *FaWRKY29* and *FaWRKY64* enhances resistance against *B. cinerea* in strawberries by triggering ABA and JA signaling. In conclusion, our results demonstrated that the knockdown of strawberry *WRKY* TF genes can increase resistance against *B. cinerea* in strawberries. *FaWRKY29* and *FaWRKY64*, as *B. cinerea* susceptible genes, could be promising candidates for developing new, CRISPR/Cas9-mediated, *B. cinerea*-resistant varieties of octoploid strawberries.

## Methods

### Plant materials and inoculations

The homozygous *Arabidopsis* T-DNA knockout lines (SALK_003729, SALK_034157, SALK_088514, SALK_100993, and SALK_201649) were obtained from TAIR (Table S1). PCR was performed with gene-specific primers and a T-DNA-specific primer to identify homozygous lines. The list of primers is available in Table S4. The *Arabidopsis* seeds with the Col-0 ecotype as control were surface sterilized with 70% EtOH followed by three times rinse with autoclaved water. Seeds were germinated on half-strength of the Murashige and Skoog (MS) medium supplemented with 1.5% (w/v) sucrose at 22°C (± 1) with a 16-h photoperiod (16 h light/8 h dark). *Arabidopsis* seedlings were transferred to soil and grown at 25°C (± 1) with a 14-h photoperiod for further inoculation test. The strawberry cultivar ‘Florida Brilliance’ was grown in the strawberry field at the Gulf Coast Research and Education Center (GCREC) in Balm, FL, USA, during the 2021–2022 winter season. Plants were managed according to the Florida strawberry industry standards. Fruits at the white stage were harvested for inoculation test. Five field isolates of *B. cinerea* (12–65, 12–221, 12–255, 12–332, and 12–355) collected from commercial strawberry fields in Florida were used in this study [67]. Each of the *B. cinerea* isolates was grown on HA agar plates (10 g l^−1^ malt extract, 4 g l^−1^ glucose, 4 g l^−1^ yeast extract, 15 g l^−1^ agar, pH 5.5) at 22°C (± 1) a 16‐h photoperiod. For *Arabidopsis* leaf inoculation with *B. cinerea*, rosette leaves collected from 4-week-old *Arabidopsis* plants were transferred to transparent square petri dishes containing 0.8% agar, inserting the petiole in the medium. The mycelia of *B. cinerea* were scraped from the surface of the isolates and spores were diluted to a concentration of 5 × 10^5^ spores ml^−1^ with potato dextrose broth. *Arabidopsis* leaves were inoculated by placing 5 µl of the spore suspension on the middle of the vein. The square petri dish containing inoculated leaves was covered with a plastic lid to keep moisture. The experiment was repeated two times and 19—24 leaves were used in each batch for each treatment. For strawberry fruit inoculation with *B. cinerea*, red fruits were prepared in plastic egg cartons five days after *Agrobacterium* infiltration with *FaWRKY29-* or *FaWRKY64*-RNAi vector. Spores were diluted to a concentration of 1 × 10^6^ spores ml^−1^ with potato dextrose broth. Strawberry fruits were inoculated by placing 20 µl of the spore suspension on the middle of the fruit. Every two egg cartons containing inoculated fruits were kept in a plastic box with a water dish to keep moisture. The experiment was repeated four times. A total of 22—30 fruits were used in each batch for each treatment. After *B. cinerea* inoculation to *Arabidopsis* leaves or strawberry fruits, disease development progress was taken pictures every day. The disease area was calculated using the ImageJ program.

### RNAi-mediated fruit transient assay

To identify ortholog sequences of *AtWRKY53* and *VvWRKY52* in octoploid strawberries, BLAST search was done using amino acid sequences from the Strawberry GARDEN (http://strawberry-garden.kazusa.or.jp) and GDR (https://www.rosaceae.org). Homologous and homoeologous copies of *FaWRKY29* and *FaWRKY64* were searched using BLAST analysis with the octoploid reference genomes from ‘Camarosa’ and ‘Florida Brilliance’. Multiple sequence alignments of *FaWRKY29* or *FaWRKY64* amino acid sequences were performed using GeneDoc. The pssRNAit program (https://www.zhaolab.org/pssRNAit/Home.gy) was utilized to design specific RNAi sequences targeting *FaWRKY29* or *FaWRKY64* [68]. *FaWRKY29*-RNAi and *FaWRKY64*-RNAi were synthesized (Genscript, NJ, USA) and cloned into pDONR^TM^221 vector and subsequently the pK7GWIWG2(i) binary vector by Gateway cloning system (Invitrogen, MA, USA).

RNAi vectors were introduced to *Agrobacterium* strain EHA105 by a freeze–thaw method [69]. For a transient assay, *Agrobacterium* was cultured at 28°C until OD_600_ of 1.0 in LB media containing 50 mg l^−1^ spectinomycin and 10 mg l^−1^ rifampicin. Bacterial pellets were resuspended with MS containing 10 mM 2-(4-morpholino) ethanesulfonic acid, 200 μM acetosyringone, and 10 mM MgCl_2_ (pH 5.5). The *Agrobacterium* with *FaWRKY29*-RNAi and *FaWRKY64*-RNAi was incubated for about 2 h in a rotary shaker (28°C, 150 rpm) before fruit infiltration. ‘Florida Brilliance’ plants were grown in the GCREC strawberry field of the University of Florida (Wimauma, FL, USA). White stage fruits were detached from plants and surface sterilized with 1% (v/v) commercial bleach, followed by rinsing three times with distilled water. About three ml of *Agrobacterium* suspension was evenly injected into fruits using a 5 ml sterile syringe until runoff. Every two egg cartons containing *Agrobacterium* infiltrated fruits were kept in a plastic box with a water dish to keep moisture. *B. cinerea* inoculation was conducted five days after *Agrobacterium* infiltration. The experiment was repeated four times, and approximately 30 fruits were used in each independent experiment.

### RNA extraction and quantitative real-time PCR

RNA extractions from fruits of mock control, EV, pK7GWIWG2(i)-*WRKY29*-RNAi, or pK7GWIWG2(i)-*WRKY64*-RNAi were conducted using the Spectrum™ Plant Total RNA Kit (Sigma–Aldrich, MO, USA) five days after *Agrobacterium* infiltration. After DNaseI treatment, the cDNA synthesis was performed using LunaScript® RT SuperMix Kit (New England Biolabs, MA, USA). The quantitative real-time PCR was performed using the Forget-Me-Not™ EvaGreen qPCR Master Mix (Biotium, CA, USA) with an internal reference gene of *FaGAPDH2* using LightCycler® 480 system (Roche, Basel, Switzerland). The primer sequences used this gene expression assay are *FaWRKY29*_F (5’-GGAGATCATTGAAGGGATGGAG-3’), *FaWRKY29*_R (5’-GAGGTCAATATCCTCTGCACTAAA-3’), *FaWRKY64*_F (5’-CACCTTCGCTAAATGGGAGT-3’), *FaWRKY64*_R (5’-TTACTTGTTTGGTCCACCGT-3’), *FaGAPDH2*_F (5’-CCCAAGTAAGGATGCCCCCATGTTCG-3’), and *FaGAPDH2*_R (5’-TTGGCAAGGGGAGCAAGACAGTTGGTAG-3’). Three biological and technical replications were used. The relative gene expression levels were calculated using the 2^−ΔΔCT^.

### Analysis of RNA sequencing and differentially expressed genes

RNA sequencing was performed with fruits (mock control, EV, pK7GWIWG2(i)-*WRKY29*-RNAi, and pK7GWIWG2(i)-*WRKY64*-RNAi) four days after *B. cinerea* infection and without the pathogen inoculation. Each RNA sample was quantified with the Qubit™ RNA high sensitivity (HS) Assay Kit (Invitrogen, MA, USA). Four individual samples were pooled equally with the same RNA concentration of 40 ng/µl. The RNA sequencing was conducted at Novogene (San Diego, CA, USA). RNA sequencing libraries were prepared with NEBNext® Ultra RNA Library Prep Kit for Illumina® (Boston, MA, USA) using the standard manufacturer’s protocol. The RNA libraries were sequenced using NovaSeq 6000 (San Diego, CA, USA) sequencer with 2 × 150 bp paired-end sequences. The Illumina RNA-Seq data are available in NCBI (BioProject number PRJNA946145). The eight cDNA libraries (mock control, EV, pK7GWIWG2(i)-*WRKY29*-RNAi, pK7GWIWG2(i)-*WRKY64*-RNAi at 0- and 4-DAI) were analyzed by FASTQC 0.11.4 (https://www.bioinformatics.babraham.ac.uk/projects/fastqc) to assess read quality. After removing adapter sequences using CLC Genomics Workbench 11.0 (https://www.qiagenbioinformatics.com/), reads with poor quality and ambiguous sequences were trimmed. The trimmed short reads were mapped to full coding region sequences of the octoploid strawberry reference genome [2] using CLC Genomics Workbench 11.0. The stringent mapping parameters were employed with a length fraction of 0.8, similarity fraction of 0.9, insertion cost = 3, deletion cost = 3, and mismatch cost = 2. Unmapped reads were discarded for further downstream analysis. Gene expression data was normalized by computing the Reads Per Million mapped reads [70]. Raw expression values were transformed by adding a constant of 5, as well as normalized by a scaling method [71]. For DEG analysis, Baggerly’s test was implemented to compare a group with another [72]. This Baggerly’s statistical test relates the mean expression value between two samples. Bonferroni correction and FDR *p*-value correction were employed [73]. The DEGs were called only if (a) the normalized fold change was ≥ threefold and (b) the *p*-value was ≤ 0.05. Pairwise evaluations between the RNA-seq samples of: EV vs. mock control, *FaWRKY29*-RNAi vs. EV, and *FaWRKY64*-RNAi vs. EV at zero and four DAI. For MapMan analysis [74], all fasta files of reference ‘Camarosa’ [2] were analyzed with the Mercator webtool (https://www.plabipd.de/portal/mercator-sequence-annotation) for Bincode mapping. The fold-change value of DEGs on each pairwise library was analyzed using MapMan [74] to identify affected metabolic pathways. Heatmap of common DEGs between *FaWRKY29*-RNAi vs. EV and *FaWRKY64*-RNAi vs. EV were visualized using TBtools software [75]. To explore the gene expression of *WRKY* TFs, RNA sequencing data were extracted from our previous study [4]. ‘Florida Brilliance' strawberry fruits were collected at six different development stages. The fruit sampling stages were categorized to Small Green (SG), Medium Green (MG), Large Green (LG), White (W), Turning Red (TR), and Red (R).

### Functional annotation of genes differentially expressed

DEGs were annotated by the BLASTX program available in the BLAST+ package [76]. The sequences were annotated with an E-value cut-off of 1e-5 against non-redundant protein database available at the genome database for Rosaceae (https://www.rosaceae.org/). The highest high-scoring segment pairs were retrieved from the corresponding database. To find the functions associated with DEGs from this study, a homology against non-redundant protein database was searched at NCBI [77]. Set enrichment analysis was performed using the singular enrichment analysis tool using the webtool AgriGO [77]. Gene ontology distribution of each pairwise library across categories was plotted using WEGO [78].

### Supplementary Information

**Additional file 1: Table S1.** List of *Botrytis* resistant *Arabidopsis* T-DNA knockout lines.

**Additional file 2: Table S2. **Total reads number and mapped rate.

**Additional file 3: Table S3.** List of DEGs. The DEGs were called if the normalized fold change was ≥ 3-fold and the *p*-value was ≤ 0.05. Pairwise evaluations between the RNA-seq samples of: EV vs. mock control, *FaWRKY29*-RNAi vs. EV, and *FaWRKY64*-RNAi vs. EV at zero and four days after *Botrytis cinerea* inoculation.

**Additional file 4: Table S4**. List of primers to detect homozygous lines in *Arabidopsis* T-DNA insertion lines.

**Additional file 5: Fig. S1.** Identification of *Arabidopsis* homozygous T-DNA insertion lines. T-DNA-specific primer and gene-specific primers were used for PCR to detect homozygous lines.

**Additional file 6: Fig. S2.** Real-time PCR was performed using RNA samples from EV, *FaWRKY29*-RNAi fruits and *FaWRKY64*-RNAi fruits collected zero days and four days after *Botrytis cinerea* inoculation.

**Additional file 7: Fig. S3. **MapMan overview map at zero days after inoculation (DAI) of *Botrytis cinerea*.

## Data Availability

The supporting datasets and supplemented materials are included within the article as additional files. RNA sequencing data for strawberry is available in NCBI SRA database with accession numbers (SRR23906067-SRR23906074 of BioProject number PRJNA946145, https://www.ncbi.nlm.nih.gov/bioproject/946145), and the data will be shared on reasonable request of the corresponding author.

## Abbreviations

ABA: Abscisic acid
BG: β–Glucosidase
BFR: Botrytis fruit rot
DAI: Days after inoculation
DEG: Differentially expressed gene
ET: Ethylene
EV: Empty vector
MS: Murashige and Skoog
OCP: Overexpressor of cationic peroxidase
PDF: Plant defensin
PME: Pectin methylesterase
Prx: Peroxiredoxin
Rboh: Respiratory burst oxidase homolog
ROS: Reactive oxygen species
SA: Salicylic acid
TAIR: The Arabidopsis information resource
TPK: Two-pore K + channel
TF: Transcription factor
WT: W ild type
