# Supplementary material for: The role of WRKY transcription factors, FaWRKY29 and FaWRKY64, for regulating Botrytis fruit rot resistance in strawberry (Fragaria × ananassa Duch.)
Source: BMC Plant Biol. 2023 Sep 11;23:420. doi: 10.1186/s12870-023-04426-1 (PMC10494375; doi:10.1186/s12870-023-04426-1)
Supplement: Supplementary file 5 — Additional file 5: Fig. S1. Identification of Arabidopsis homozygous T-DNA insertion lines. T-DNA-specific primer and gene-specific primers were used for PCR to detect homozygous lines. [file 12870_2023_4426_MOESM5_ESM.pptx]

## Slide 1
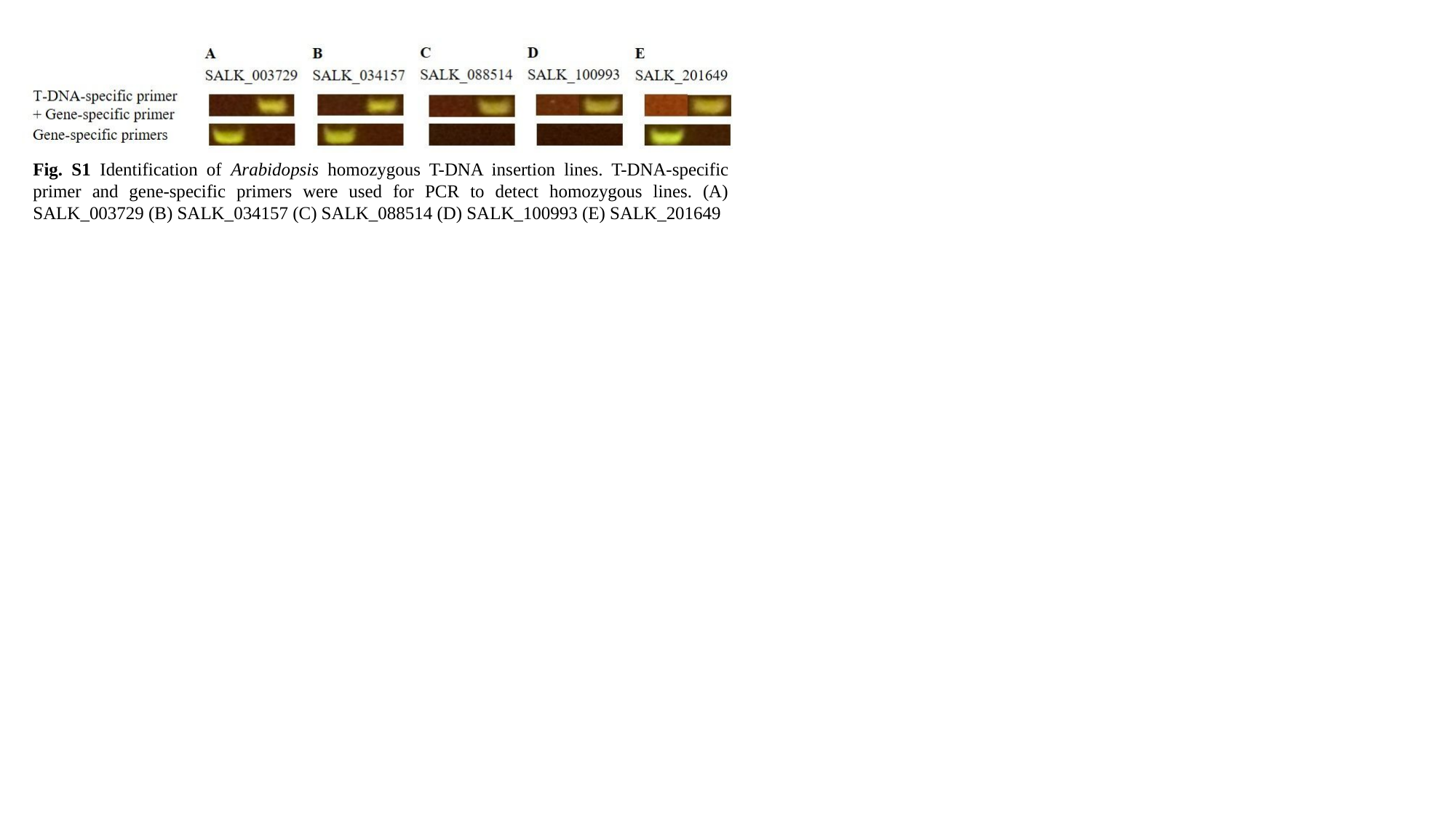

Fig. S1 Identification of Arabidopsis homozygous T-DNA insertion lines. T-DNA-specific primer and gene-specific primers were used for PCR to detect homozygous lines. (A) SALK_003729 (B) SALK_034157 (C) SALK_088514 (D) SALK_100993 (E) SALK_201649
