# Supplementary material for: The role of WRKY transcription factors, FaWRKY29 and FaWRKY64, for regulating Botrytis fruit rot resistance in strawberry (Fragaria × ananassa Duch.)
Source: BMC Plant Biol. 2023 Sep 11;23:420. doi: 10.1186/s12870-023-04426-1 (PMC10494375; doi:10.1186/s12870-023-04426-1)
Supplement: Supplementary file 6 — Additional file 6: Fig. S2. Real-time PCR was performed using RNA samples from EV, FaWRKY29-RNAi fruits and FaWRKY64-RNAi fruits collected zero days and four days after Botrytis cinerea inoculation. [file 12870_2023_4426_MOESM6_ESM.pptx]

## Slide 1
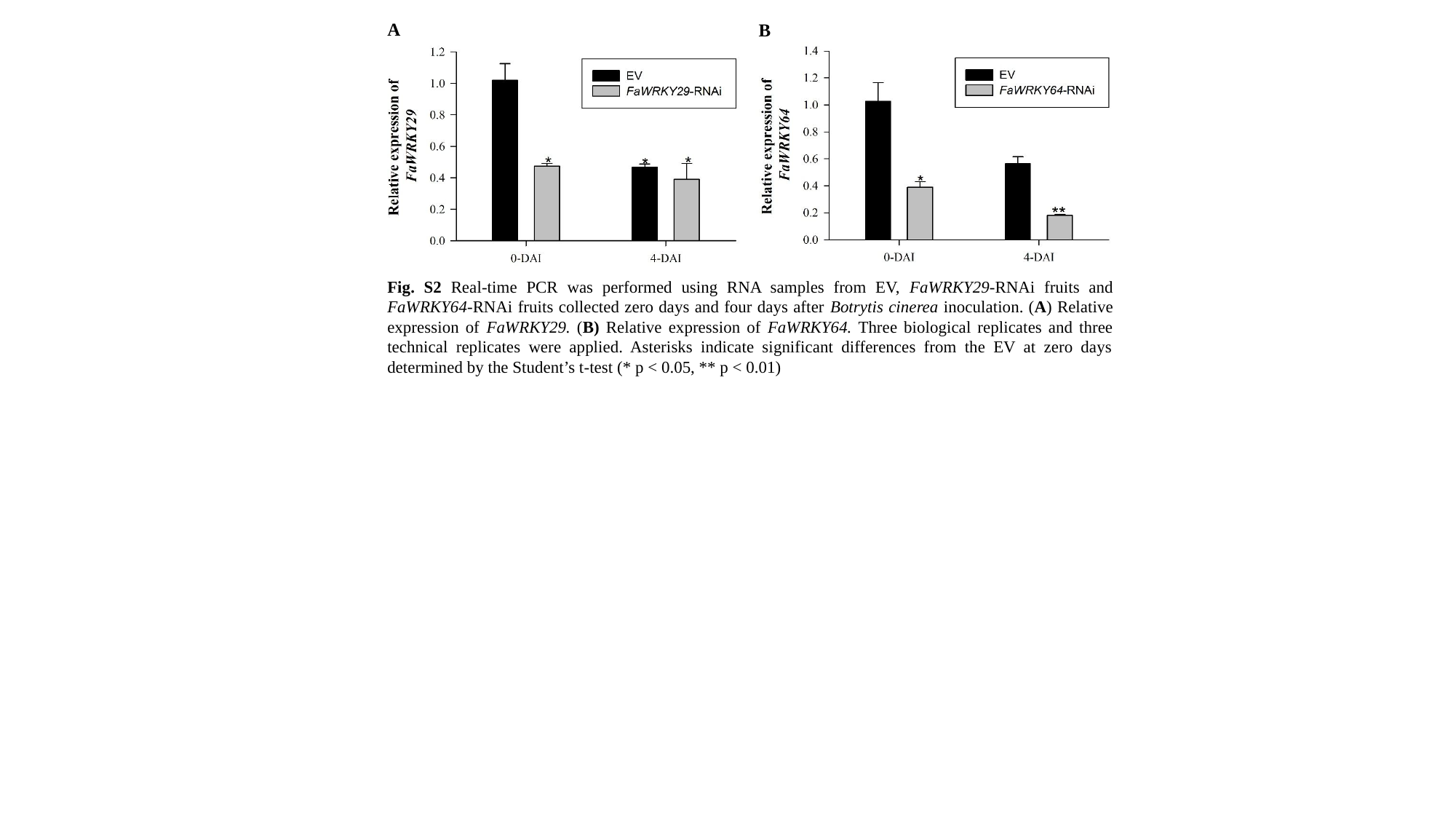

A
B
Fig. S2 Real-time PCR was performed using RNA samples from EV, FaWRKY29-RNAi fruits and FaWRKY64-RNAi fruits collected zero days and four days after Botrytis cinerea inoculation. (A) Relative expression of FaWRKY29. (B) Relative expression of FaWRKY64. Three biological replicates and three technical replicates were applied. Asterisks indicate significant differences from the EV at zero days determined by the Student’s t-test (* p < 0.05, ** p < 0.01)
