# Supplementary material for: The role of WRKY transcription factors, FaWRKY29 and FaWRKY64, for regulating Botrytis fruit rot resistance in strawberry (Fragaria × ananassa Duch.)
Source: BMC Plant Biol. 2023 Sep 11;23:420. doi: 10.1186/s12870-023-04426-1 (PMC10494375; doi:10.1186/s12870-023-04426-1)
Supplement: Supplementary file 7 — Additional file 7: Fig. S3. MapMan overview map at zero days after inoculation (DAI) of Botrytis cinerea. [file 12870_2023_4426_MOESM7_ESM.pptx]

## Slide 1
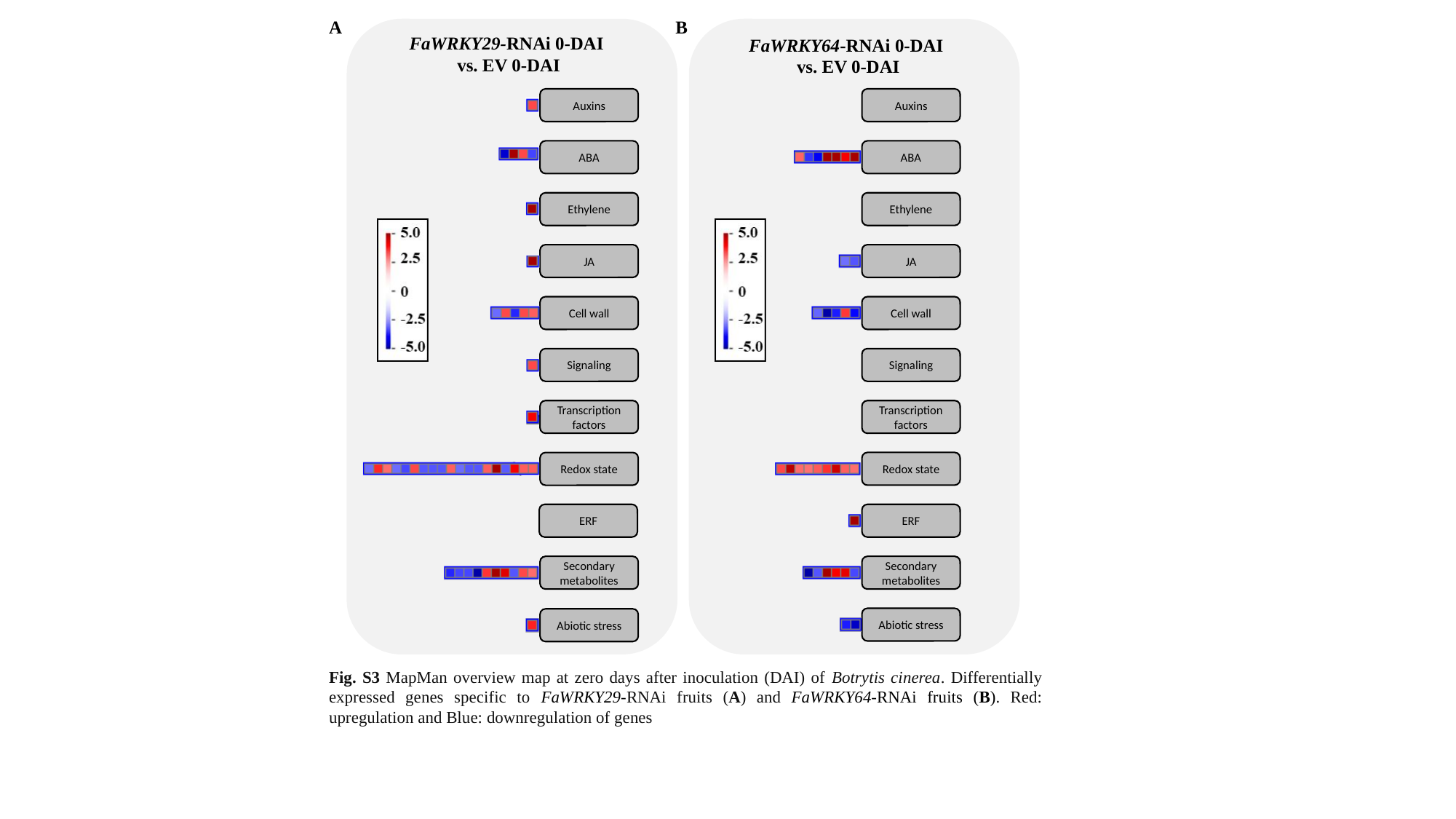

A
FaWRKY29-RNAi 0-DAI
vs. EV 0-DAI
Auxins
ABA
Ethylene
JA
Cell wall
Signaling
Transcription factors
Redox state
ERF
Secondary metabolites
Abiotic stress
B
FaWRKY64-RNAi 0-DAI
vs. EV 0-DAI
Auxins
ABA
Ethylene
JA
Cell wall
Signaling
Transcription factors
Redox state
ERF
Secondary metabolites
Abiotic stress
Fig. S3 MapMan overview map at zero days after inoculation (DAI) of Botrytis cinerea. Differentially expressed genes specific to FaWRKY29-RNAi fruits (A) and FaWRKY64-RNAi fruits (B). Red: upregulation and Blue: downregulation of genes
